# The Osteosarcoma Microenvironment: A Complex but Targetable Ecosystem

**DOI:** 10.3390/cells9040976

**Published:** 2020-04-15

**Authors:** Isabelle Corre, Franck Verrecchia, Vincent Crenn, Francoise Redini, Valérie Trichet

**Affiliations:** 1 INSERM, Nantes University, UMR1238 Phy-Os “Bone Sarcomas and Remodeling of Calcified Tissues”, F-44035 Nantes, France; 2 CNRS GDR3697 MicroNit, F-37044 Tours, France; 3 Department of Orthopedic, Nantes Hospital, CHU Hotel-Dieu, F-44035 Nantes, France

**Keywords:** Osteosarcoma, microenvironment, bone, stromal cells, vascular cells, targeted therapies, extracellular vesicles, multi-kinase inhibitors

## Abstract

Osteosarcomas are the most frequent primary bone sarcomas, affecting mainly children, adolescents, and young adults, and with a second peak of incidence in elderly individuals. The current therapeutic management, a combined regimen of poly-chemotherapy and surgery, still remains largely insufficient, as patient survival has not improved in recent decades. Osteosarcomas are very heterogeneous tumors, both at the intra- and inter-tumor level, with no identified driver mutation. Consequently, efforts to improve treatments using targeted therapies have faced this lack of specific osteosarcoma targets. Nevertheless, these tumors are inextricably linked to their local microenvironment, composed of bone, stromal, vascular and immune cells and the osteosarcoma microenvironment is now considered to be essential and supportive for growth and dissemination. This review describes the different actors of the osteosarcoma microenvironment and gives an overview of the past, current, and future strategies of therapy targeting this complex ecosystem, with a focus on the role of extracellular vesicles and on the emergence of multi-kinase inhibitors.

## 1. Introduction

Osteosarcomas (OSs) are the most common primary malignant bone sarcomas, with a bimodal age distribution. The highest incidence is in children and adolescents (median of age of 18), with a second smaller peak of incidence in elderly individuals over 60 years. Worldwide, the incidence of OS is around one to three cases annually per million individuals [1]. These tumors develop mainly in the long bones (femur, tibia, humerus), close to the growth plate in the bone metaphysis, and less frequently in the skull, jaw, and pelvis. OSs are characterized by the presence of transformed osteoblastic cells producing osteoid matrix. Nevertheless, the precise identity of the cell at the origin of the tumor remains unknown. Evidence supports the idea of an origin of OS in mesenchymal stem/stromal cells (MSCs) and/or in more committed osteoblastic precursors [2,3]. Since the introduction of chemotherapies to treat OS the late 70s, patients diagnosed with OS receive a neo-adjuvant treatment followed by a post-surgery adjuvant therapy with a cocktail of chemotherapies, i.e., high-dose methotrexate (12 g/m^2^), etoposide, and ifosfamide for children and young adults (<25 years) in the French OS2006/sarcome-09 study [4], or other protocols combining doxorubicin, cisplatin, and ifosfamide with or without high-dose methotrexate [5,6,7]. With these therapeutic regimens, the 5 year survival has reached 78% for children and young adults with localized disease, but still remains at only 20% in patients with metastasis at diagnosis or in relapse [1,4]. Moreover, in the last 40 years, survival has not notably improved for patients without metastases and has not improved at all for metastatic patients [8]. Therefore, improving therapy for OS remains a constant and major goal for many worldwide research and clinical groups.

A major characteristic of OSs tumors is their heterogeneity, both at the intra-tumoral level and also between individuals. Therefore, the common genomic initiating biological processes driving osteosarcomagenesis are still not identified. The complexity of the somatic genome of OS is a major cause of intra-tumoral heterogeneity, characterized by chromosomal aneuploidy, alteration of genes by mutation and/or variation of copy number, genomic instability featured by massive rearrangement through chromotripsis, and the presence of patterns of localized hypermutated regions, named kataegis [9]. A small set of genes has been found to be recurrently mutated in OS (*TP53*, *RB*, *MDM2*, *ATRX*, and *DLG2*) [10]. Recently, a subset of OSs was described with genomic alterations in genes of the DNA repair pathways, reminiscent of *BRCA1/2*-deficient tumors [11]. Several inherited syndromes such as Li–Fraumeni, Rothmund–Thomson, Werner, Bloom, and retinoblastoma familial cancers have also been associated with a predisposition to developing OS [9]. Nevertheless, in the vast majority of cases (95%), OSs appear as sporadic events. Overall, poorly defined oncogenic events associated with high cellular heterogeneity of tumor cells make the development of molecular targeted therapies devoted exclusively to tumor cells difficult.

Bone sarcomas, and in particular OSs, grow in the bone microenvironment, a very specialized, complex, and highly dynamic environment composed of bone cells (osteoclasts, osteoblasts, osteocytes), stromal cells (MSCs, fibroblasts), vascular cells (endothelial cells and pericytes), immune cells (macrophages, lymphocytes), and a mineralized extracellular matrix (ECM). In physiological conditions, a coordinated and fine-tuned orchestrated activity of bone, vascular, and stromal cells ensures bone homeostasis through intense paracrine and cellular communications. According to Paget’s theory [12], tumor cells find in this microenvironment a fertile soil to seed and manage to highjack bone physiological pathways to their advantage in order to survive and grow. Cross-talk between OS and the bone microenvironment involves numerous environmental signals, induced by multiple cytokines, chemokines, and soluble growth factors [13], but also conveyed by extracellular vesicles (EVs), considered today to be effective vectors of communication between cells [14].

In OS, the difficulty of designing and validating new therapies rests on two levels of complexity: first, a high heterogeneity in tumor cells with no evident targetable event, and second, an active and reacting microenvironment composed of active cells, interconnected and intensively communicating through paracrine secretion of soluble factors and EVs. In this review, we describe the different actors of the OS microenvironment in the context of their complex interaction with tumors cells. We also discuss the past, current, and future therapeutic strategies, regarding the complex ecosystem of OS, with a focus on the emergence of multi-kinase inhibitors (MKI) that target tumor cells and the cells of their microenvironment, and on the role of EVs as essential conveyors of information in bone sarcoma biology.

## 2. OS-Induced Bone Remodeling

### 2.1. Osteoclasts and Osteolysis

OS development is associated with para-tumor osteolysis, causing frequent painful bone fragility at the time of the detection of OS in patients. OS aggressiveness has been associated with osteolysis markers in a few clinical cases [15]. Notably, the binding of the soluble molecule Receptor Activator of Nuclear Factor kappa B Ligand (RANKL), alias TNFSF11, to its receptor (RANK), mainly regulates osteolysis through paracrine regulation. RANKL is produced by osteoblasts and osteocytes in the bone environment [16], while RANK is expressed on the cell surface of osteoclast precursors [17]. In OSs, osteoclast activity leads to a vicious cycle between OS cell proliferation and bone degradation, leading to the release of pro-tumor factors such as insulin-like growth factor 1 (IGF1) or transforming growth factor-β (TGF-β) from the bone matrix [13,18]. However, clinical trials using monoclonal antibody therapy to block the IGF receptor tyrosine kinase in patients with OS showed limited and unpredictable response rates, leading to the cessation of this therapy [19].

The link of osteolysis in the vicious cycle observed in OS has been demonstrated in preclinical studies, using either chemical inhibitors (mainly zoledronic acid, ZOL) [20,21] or RANKL receptor competitors (including osteoprotegerin (OPG) [22], RANK-Fc [23]), or *RANKL* silencing [24]. Thus, osteolysis inhibition became an attractive therapeutic target in combination with chemotherapeutics to treat OS. However, initiated on the basis of promising preclinical studies, OS2006, a Phase III clinical trial combining ZOL with chemotherapy and surgery gave very disappointing results, with no improvement but slightly worse therapeutic results [25]. Despite the fact that ZOL has also been described in vitro to have a direct effect on OS cells, its efficacy against OS primary growth and pulmonary metastasis remains controversial [26].

Direct implication of osteoclast activity in OS development and progression in patients is still difficult to decipher. Indeed, a loss of osteoclasts was associated with increased metastasis in a preclinical model of OS [27], while co-injection of pre-osteoclasts with human OS cells had no effect on OS local growth and lung metastases in nude mice [28]. Denosumab, an antibody directed against RANKL, efficiently inhibits osteoclast activity and is currently used to treat bone loss in bone metastasis, multiple myeloma, or giant cell tumors. However, no clinical results have been reported to date for denosumab in OS patients, except in combination with the MKI sorafenib for one patient [29,30]. Even following a more specific targeting of RANKL, denosumab does not have differentiated action towards different cell types. Indeed, the RANKL/RANK pathway is involved not only in osteoclasts, but also in many other cells of the tumor environment, including osteoblasts, stromal cells, immune cells (T and B lymphocytes, dendritic cells), and endothelial cells.

**Figure 1:**
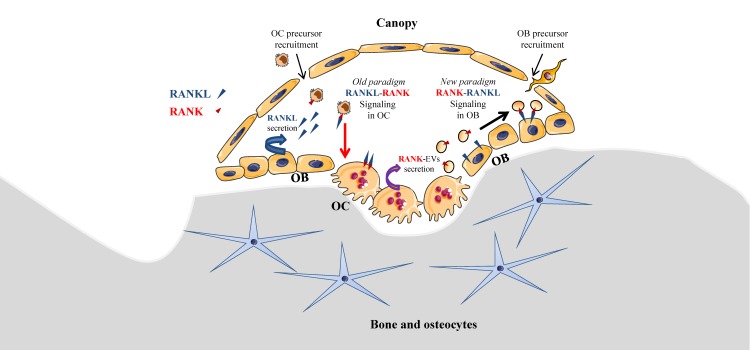
RANK–RANKL interactions under the canopy of bone remodeling compartments: old and new paradigms. The canopy is generated by bone lining cells and insulates a basic multicellular unit with osteoclasts (OC) and osteoblasts (OB). OC and OB precursors are recruited under the canopy, from respectively bone marrow- and blood stream-supplied hematopoietic stem cells and bone marrow-issued mesenchymal stromal cells. In the old and well-demonstrated paradigm, RANKL secreted by OB induces OC differentiation through RANK intracellular signaling (RANKL-RANK), while a new paradigm proposes a reverse signaling through RANKL intracellular signaling (RANK-RANKL) mediated by RANK-bearing extracellular vesicles EVs from OC [32]. RANK: receptor activator of nuclear factor kappa-B; RANKL: RANK ligand.

Local coupling between bone resorption and formation is essential to preserve bone density and should occur in basic multicellular units, including osteoclasts and osteoblasts, which are covered by bone lining cells forming a canopy, as originally described by Lassen et al. [31]. Under the canopy, RANKL secreted by osteoblasts induces osteoclast differentiation, as described in a well-demonstrated paradigm. Interestingly, a new paradigm model of intercellular communication of osteoclasts towards osteoblasts may be relevant (Figure 1), as it was recently reported that mature osteoclasts were able to produce EVs bearing RANK, allowing interaction with RANKL on osteoblasts [32]. RANK-bearing EVs were initially identified in mouse primary osteoclasts and precursors derived from bone marrow [33]. Recently, Ikebuchi et al. effectively demonstrated that RANK-bearing EVs issued from mouse mature osteoclasts were able to interact with RANKL-expressing osteoblasts, and therefore to induce osteoblastic differentiation coupled with bone formation involving RUNX2 signaling [32]. RANKL-reverse signaling in osteoblasts was demonstrated using RANK-masking on EVs and by creating a mutant mouse model *Rankl^P29A^*, where RANKL intracellular signaling domain was suppressed. Consequently, RANK–RANKL interaction appears to be bi-directional, dual, and complementary in the coupling of bone resorption and formation: RANK transduction on osteoclasts and precursors activates osteolysis, while RANKL transduction on osteoblasts and precursors activates osteogenesis.

**Figure 2:**
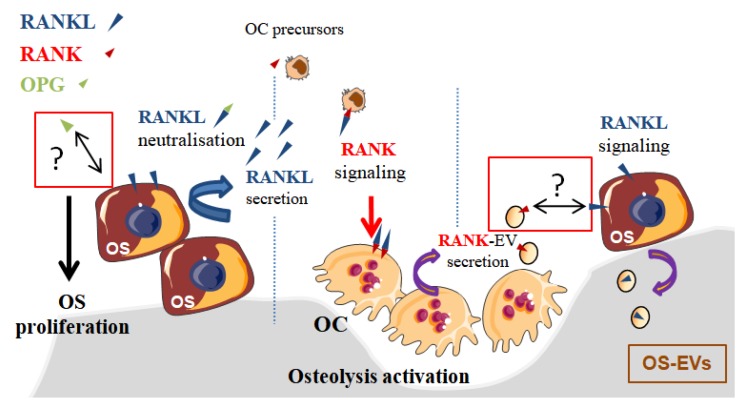
Proposed model of OPG/RANK–RANKL interactions in osteosarcoma (OS). RANK transduction induces the differentiation of osteoclasts (OC), leading to osteolysis, which in turn activates tumor cell proliferation, described as the vicious cycle. OPG is a decoy form of RANK, binding and neutralizing RANKL. Additionally, OPG increases proliferation of RANKL-expressing OS cells following its binding to an unknown receptor [38], possibly RANKL. The reverse signaling of RANKL, described recently in osteoblasts [32], could be also induced in OS cells. To the same extend, OS cells expressing RANKL could be activated by RANK-extracellular vesicles (EVs) produced by OC. RANK: receptor activator of nuclear factor kappa-B; RANKL: RANK ligand; OPG: osteoprotegerin.

In the context of OS, bone remodeling is linked to a vicious cycle between osteoclasts and tumor cells [22], which is established through the release of growth factors from the degraded bone matrix. Nevertheless, this vicious cycle may be additionally enhanced by EVs secreted by osteoclasts and OS cells [34]. Indeed, EVs secreted by OS cells were able to enhance osteolysis, while RANK-EVs secreted by osteoclasts may activate RANKL expressed on OS cells [35], suggesting a possible RANK–RANKL reverse signaling in OS, as previously described in normal bone physiology [32]. In one retrospective clinical study involving 40 patients, RANKL expression was observed in 75% of OS biopsy samples and its high expression level was correlated to a poor patient outcome [36]. Branstetter et al. [37] detected RANKL in 68% of human OSs, but only 37% OS samples showed more than 10% of tumor cells expressing RANKL. The same year, it was reported that the proliferation of RANKL-expressing OS cell lines was increased through transduction signaling involving AKT and ERK activation when cells were exposed to OPG [38]. One could hypothesize that RANKL expressed on the surface of OS cells could have been activated by OPG, as this protein is the decoy and soluble form of RANK that binds RANKL (Figure 2). Nevertheless, this pro-proliferative effect of OPG was believed to be independent of RANKL because soluble RANK did not induce similar effects. Thus, it was proposed that OPG’s pro-proliferative effect was mediated by an unknown receptor. In regard to the innovative identification of the RANKL reverse signaling as described above (Figure 1 and Figure 2) [32], RANKL activation in OS cells should be revisited, as RANK-EVs released by osteoclasts may have an unexpected role in OS through a possible RANK–RANKL reverse signaling in OS cells.

### 2.2. Osteoblasts and Bone Formation

Primary bone tumors have potent local influences on bone and the clinical consequences of these influences can be devastating. OS is characterized by the formation of osteoid matrix surrounding anaplastic tumor cells [39,40], and it can stimulate the formation of various bone structures, such as Codman’s triangles or bone spines, designed as the sunburst periosteal reaction. The sunburst pattern of bone is due to new layers of collagen fibers stretching out perpendicularly to the bone. This process is mainly due to a deregulation of bone remodeling and in part to the activity of non-tumor osteoblasts, as observed in mouse OS models.

**Figure 3:**
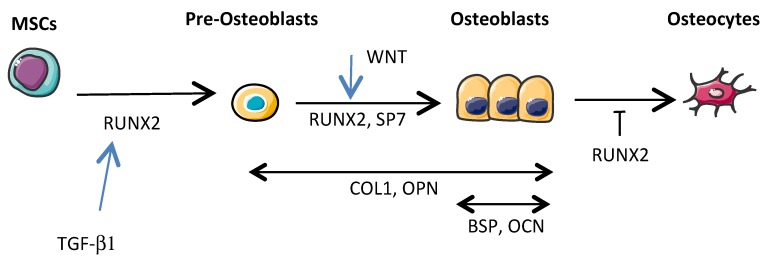
Osteoblastic differentiation from mesenchymal stem/stromal cells (MSCs) to osteocytes. Transcription factor RUNX2 promotes MSC commitment toward the osteoblastic lineage at the early stages while repressing maturation in osteocytes. SP7 allows the differentiation of pre-osteoblasts into functional mature osteoblasts. RUNX2 induces expression of genes coding for expression of collagen type 1 (COL1), osteopontin (OPN), bone sialoprotein (BSP) and osteocalcin (OCN) proteins. Transforming growth factor-β (TGF-β1) and WNT stimulate early stages of osteoblastic differentiation.

Osteoblastic progenitors are MSCs mainly present in the bone marrow, and more specifically multipotent skeletal stem cells (MSSCs), which are a subset of MSCs that were recently identified [41]. Under the control of different specific transcription factors, MSCs are able to differentiate into osteoblasts, chondroblasts, myoblasts, and adipocytes, while MSCCs differentiate into osteoblasts and chondroblasts, but not into myoblasts and adipocytes. However, there is not yet evidence indicating that either MSCs or MSSCs are the most important cells in the pathogenesis of OS. Current knowledge on osteoblastogenesis is based on MSC rather than MSSC differentiation. Briefly, RUNX2 and Osterix or SOX9 transcription factor expression leads to MSC differentiation, respectively towards the osteoblastic and chondroblastic lineages [42]. The differentiation of MSCs into mature osteoblasts involves a complex series of proliferation and differentiation steps (Figure 3). Briefly, RUNX2 (also known as CBFA1) is a transcriptional factor that binds a consensus site, called OSE2, present along the proximal promoters of many genes including those of the α1 chain of type I collagen (*COL1A1*), bone sialoprotein (*BSP*), osteocalcin (*OCN*), and osteopontin (*OPN*) [43,44]. RUNX2 is crucial for the early steps of MSC differentiation into pre-osteoblasts and to maintain osteoblastic function, while Osterix (also known as SP7) is involved in osteoblastic differentiation mainly downstream of RUNX2 by allowing the differentiation of pre-osteoblasts into functional mature osteoblasts [45]. Upstream of those transcriptional factors, a signal transduction cascade has to be activated by cytokines or growth factors such as TGF-βs, fibroblast growth factors (FGFs), or wingless-type MMTV integration site family members (WNTs). Most of these cytokines or growth factors are implicated in OS development.

The TGF-β family comprises at least 30 members in humans [46]. The role of TGF-βs during bone remodeling is complex. Regarding the mesenchymal osteoblastic lineage, TGF-β1 favors bone formation by stimulating the proliferation and migration of MSCs during the early stages of osteoblastogenesis [47,48]. In contrast, during the late stages of osteoblastogenesis, TGF-β1 inhibits the differentiation of MSCs into osteoblasts and the mineralization of mature osteoblasts in culture [49]. Interestingly, TGF-β1 is mainly implicated in OS development during either primary tumor growth or metastatic progression [50]. Blocking TGF-β activity in OS cells by SMAD7 overexpression has decreased primary tumor growth by affecting the relationships between tumor cells and non-tumor cells [51].

FGFs are also key regulators of skeletal development [52]. For example, FGF2 is important for the proliferation and maturation of pre-osteoblasts, while FGF18 is essential for mature formation of osteoblasts. Therefore, FGF receptors are receptor tyrosine kinases that may represent a therapeutic target in OS patients [53]. Indeed, Weekes et al. reported an important decrease of lung metastases upon using the inhibitor AZD4547 to block FGF receptor signaling following OS induction in mice [54].

WNTs are a family of 19 secreted glycoproteins. The binding of a WNT ligand (i.e., WNT1, WNT3a) to a frizzled (FZD) receptor, and its co-receptor LRP5/6 activates the canonical WNT pathway [55]. Activation of the WNT signaling cascade leads to the promotion of bone formation and suppression of bone resorption, leading to a balance in bone remodeling [56]. Interestingly, a monoclonal antibody against the WNT signaling inhibitor dickkopf-1 inhibited OS metastasis in a preclinical model of OS [57].

Evidence is thus emerging for a role of osteoblasts in tumor growth in bone. Osteoblasts directly regulate bone matrix synthesis by their own secretome and indirectly regulate bone resorption through the release of RANKL, which binds RANK on osteoclast precursors as previously presented (Figure 1). Additionally, RANK is expressed on MSCs and is downregulated during osteoblastogenesis. Intriguingly, Branstetter et al. did not detect RANK expression on tumor cells into OS samples [37]. Nevertheless, one might address the importance of RANK signaling in OS cells, which derived from cells committed in differentiation pathway between MSCs or pre-osteoblasts towards mature osteoblasts [58]. In this context, Navet et al. investigated the role of RANK overexpression in OS cell lines and during OS development in immune-deficient mice [59]. Activation of the RANKL–RANK pathway in these OS cell lines did not change cell proliferation or migration, nor tumor growth in vivo. Such results suggest that RANK activation in OS cells is not involved in tumor growth. However, RANK-overexpressing OS cells induced a significant increase of lung metastases that was prevented with an antibody directed against RANKL. In another study [23], whole body deletion of RANKL proteins prevented OS development and lung metastases in genetically predisposed mice while, in contrast, Rank deletion in osteoblasts did not change OS burden, nor lung metastasis. RANKL–RANK pathway activation does not seem to be directly implicated in OS development, but can be indirectly involved in OS progression. Implication of a potential RANKL reverse signaling in OS cells has not been tested in these studies, but it would be interesting now to take into account the implication of RANKL transduction on osteoblasts [32] (Figure 1). Antibodies against RANKL and the whole-body deletion of RANKL could disrupt the coupling between bone resorption and formation and modify the progression of OS by inhibiting the transduction of RANKL on osteoblasts and on OS cells expressing RANKL (Figure 2).

## 3. MSCs in OS Microenvironment

### 3.1. MSCs as Sensors and Modulators of OS Microenvironment

In OS, interactions between the tumor parenchyma and the non-tumor stroma are required during tumor development and metastatic progression [60]. MSCs are sensors of their microenvironment as they express multiple growth factors and chemokine signaling receptors. They also modulate their microenvironment as they secrete components of ECM and a large variety of mitogenic growth factors, cytokines, chemokines, and metalloproteinases (MMPs) [61]. Consequently, MSCs have autocrine and paracrine trophic properties, as their secreted growth factors stimulate cell division and differentiation of MSCs, osteoblasts, and endothelial cells [62]. Furthermore, MSCs also secrete chemokines (C-C motif) ligand 5 (CCL5), stromal derived factor 1 SDF-1 or (C-X-C motif) chemokine 12 CXCL12, interleukin 6 (IL-6), and growth factor vascular endothelial growth factor (VEGF), known, among others, to promote OS growth, metastasis spread, and angiogenesis (reviewed in Reference [63]).

**Figure 4:**
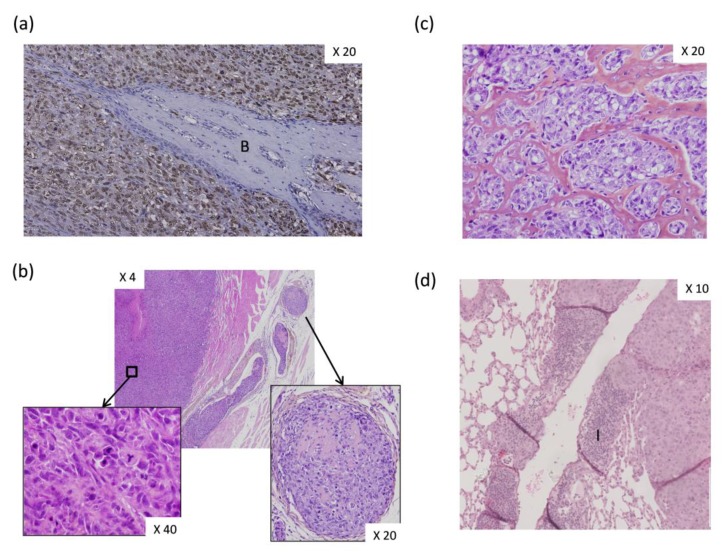
Histological analysis of experimentally induced osteosarcoma (OS) in athymic mice. Human OS-inducing cells (MNNG-HOS cells, CRL-1547, from American Type Culture Collection) were injected either alone (**a**,**b**) or co-injected with OS-derived stromal cells (OSDCs) (**c**,**d**) at tibial sites of athymic mice. Tumor samples were fixed in 10% buffered formaldehyde, embedded in paraffin wax, sectioned, and stained. Magnifications are indicated. (**a**) Human OS cells (brown nuclei) were distinguished from mouse cells (blue nuclei) by in situ hybridization using the human-specific repetitive Alu sequence [65]. Bone (B) spine lined by mouse cells was observed. (**b**) MNNG-HOS-induced tumor section was stained with hematoxylin–eosin–safran solution (HES). Tumor developed in muscle and appeared as an undifferentiated pleomorphic sarcoma (magnification in left panel), while osteoid matrix was observed only in intra-vascular tumor emboli (magnification in right panel). (**c**) Following co-injection with OSDC [64], MNNG-HOS-induced tumor was visible with abundant osteoid matrix surrounding anaplastic tumor cells. Tumor section was stained with HES. (**d**) Image of lung metastasis developed from co-injection of MNNG-HOS and OSDCs at paratibial site. Remarkable and non-usual immune cell infiltration (I) was observed in the vessel wall.

In a previous study, we co-injected OS-associated stromal cells, also named OS-derived cells (OSDCs), with N-methyl-N′-nitro-N-nitrosoguanidine (MNNG)-HOS OS cells, derived from a human OS, in nude mice [64]. We found several new observations at the histological level. First, the primary OS induced by MNNG-HOS cell injection near the tibia of nude mice appeared as an undifferentiated pleiomorphic sarcoma inducing bone spines (Figure 4a), while some osteoid matrix could be observed in intra-vascular tumor emboli (Figure 4b). Subsequently, following the co-injection of MNNG-HOS cells with OSDCs in nude mice, it was surprising to observe an abundant osteoid matrix (Figure 4c), usually not observed in the MNNG-HOS mouse model, but detected as a hallmark of OS tumor in patients. The second change involved a huge infiltration of peripheral blood mononuclear cells in vessel walls surrounding OS metastases in the lungs (Figure 4d). However, we do not know how MSCs influence OS cells and how MSC-educated OS cells may in turn influence their surrounding cells, leading to more osteoid matrix and higher immune infiltration. In this context, Pietrovito et al. described how MSCs in contact with OS cells gained a cancer-associated fibroblast phenotype and, in turn, how OS-activated MSCs promoted OS cell motility, invasiveness, and transendothelial migration [60]. OS-activated MSCs increased the secretion of monocyte chemoattractant protein (MCP)-1 (alias CCL2), growth-regulated oncogene (GRO)-α (also known as CXCL1), and IL-6 and -8.

MSCs and MSC-derived osteoblasts secrete components of ECM and MMPs. Tumor infiltration of immune cells is tightly dependent on ECM plasticity and proteolysis [66]. As demonstrated by Nicolas-Boluda et al. using imaging technology, T cells may cross the blood vessels but remain trapped in the stroma surrounding tumor nodules [67]. Consequently, T cells do not secrete MMPs allowing matrix lysis, and they then cannot progress alone between dense and tight fibers. Dynamic immune cell infiltration has not yet been imaged in OS and was not yet related to fiber density and MMPS in surrounding ECM; however, induced proteolysis could be a key to achieving success of any immune-cell therapy in OS.

### 3.2. MSCs as Donors and Acceptors of Extracellular Vesicle Cargo in OS Microenvironment

**Figure 5:**
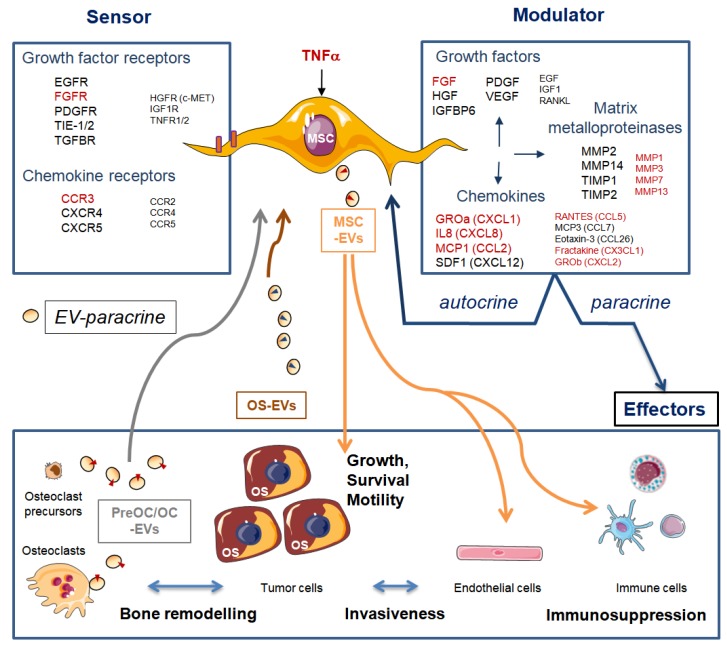
Sensing and modulating roles of mesenchymal stem/stromal cells (MSCs). MSCs constitutively express many mitogenic growth factors, chemokines, and matrix metalloproteinases at various levels. High/intermediate and low levels are represented by large or small font size, respectively. MSCs respond to tumor necrosis factor alpha (TNFα) by increasing expression (indicated in red) of some growth factor receptors (GFR), growth factors (GF), chemokine receptors (CR), chemokines, interleukins (IL), and matrix metalloproteinases (MMP) [61]. MSCs have autocrine and paracrine trophic properties, as all of these growth factors are able to act on several effectors of the tumor ecosystem (tumor, bone cells, endothelial cells, and immune cells). MSCs can also secrete extracellular vesicles (EVs) that convey diverse contents (see text for details), and they can receive information through EVs from bone pre-osteoclasts (pre-OC) and osteoclasts (OC) and tumor cells. EGFR: epithelial GFR; VEGFR: vascular endothelial GFR; TIE-1: angiopoietin receptor; TGFBR: transforming growth factor beta receptor; TIMP: tissue inhibitors of MMP; CXCL: chemokine (C-X-C motif) ligand; CCL: chemokine (C-C motif) ligand; GROα/β: growth-regulated oncogene alpha/beta (also known as CXCL1/2); MCP1/3: monocyte chemoattractant protein (also known as CCL2/7); SDF1: stromal cell-derived factor (also known as CXCL12); RANTES: Regulated on Activation Normal T Cell Expressed and Secreted (alias CCL5).

The secretome of MSCs contains bioactive EVs, which may explain some well-known but unraveled therapeutic roles of MSCs, including their role in bone regeneration [62]. MSC-secreted EVs were shown to bear tumor supportive microRNAs and proteins, but also metabolites such as lactate and glutamate [68]. A previous study showed metabolic cooperation between OSDCs and OS cells through lactate efflux by MSCs and its uptake by OS cells [69]. This metabolic cooperation can be amplified by lactate released in EVs secreted by OSDCs. Moreover, MSC-secreted EVs were shown to increase OS cell survival and migration, especially under stress induced by serum starvation [70] (Figure 5).

Gebraad et al. isolated EVs from lipopolysaccharide-activated monocytes and RANKL-activated osteoclasts, both EVs bearing RANK, and observed EV effects on gene expression and protein secretion of MSCs [71]. EVs secreted by activated monocytes, but not those secreted by osteoclasts, promoted the expression of MMP genes such as MMP3 and MMP1, and secretion of the chemokine CXCL5. The same group showed that OS-secreted EVs on MSCs promoted expression of *MMP1*, *VEGF-A*, and intercellular adhesion molecule (*ICAM1*) genes, which in turn may stimulate not only bone remodeling, but also tumor angiogenesis and metastasis [72].

EVs secreted by OS induced hypomethylation on long interspersed elements (LINE-1) of MSCs [72]. Hypomethylation on LINE-1 retro-transposons correlates with chromosomal instability and may drive oncogenetic effects [73]. Genomic instability in MSCs links back to the fact that MSCs have been proposed as cells of origin for high-grade OS [74], which is characterized by complex genetic rearrangements [64]. Large genomic studies have demonstrated that high-grade OS presents one of the highest levels of chromosomal instability associated with hyper-mutations. However, the molecular mechanisms initiating and underlying such huge chromosomal rearrangements are still unknown in the pathogenesis of OS [75,76].

Endo-Munoz et al. observed that some metastatic OS cell lines, including the KHOS cell line (CRL-1544 from American Type Culture Collection), produce high level of the protease urokinase plasminogen activator (uPA) and of its receptor (uPAR), leading to an autocrine loop of activation [77]. They showed that uPAR activation drives ERK phosphorylation and stimulates in vitro migration and in vivo propagation of OS cells. Moreover they demonstrated that KHOS cells produce EVs bearing uPA, which may be biologically active. Additionally, they identified a paracrine uPA axis involving stromal cells in mouse bone marrow explants cultured for 6 days in the presence of macrophage colony-stimulating factor (M-CSF or CSF-1). These mouse stromal cells were not characterized by surface marker analysis, but likely contained a large fraction of monocytes and/or hematopoietic stem cells. The implication of MSCs in this uPA paracine activation of OS cells was not shown, whereas uPA is a well-known activator of MSCs mobilization and transendothelial migration during tissue repair [78]. Above all, they identified uPA inhibition as a promising therapeutic strategy to prevent metastatic spread in OS patients. In a mouse model of OS, OS-secreted EVs bearing TGF-β induced a pro-tumorigenic and pro-metastatic phenotype of MSCs through increased IL-6 secretion and activation of STAT3 signaling pathways in OS cells [79]. This study showed how MSCs that have been conditioned or educated by OS-EVs may favor a tumor’s progression in its microenvironment.

MSC-secreted EVs may be also involved in revascularization following treatment-induced hypoxia. Indeed, EVs released by hypoxia-treated MSCs have increased angiogenesis through activation of the protein kinase A in human umbilical vein endothelial cells (HUVECs) [80]. EVs from MSCs have been described as able to act on endothelial cells to deliver angiogenic signals [81], especially in hypoxic and ischemic conditions [82,83]. In the bone context, pro-angiogenic effects of MSC-derived EVs have been shown in an osteonecrosis model [84,85]. Angiogenic properties of MSC-EVs have not been explored yet in OS. EVs are able to transfer growth factors, cytokines, and nutrients, as presented in this review, but can also transfer also nucleic acids. DNA can be expelled from cells by EV release during drug-induced senescence in order to maintain cellular homeostasis [86], whereas microRNAs contained in EVs can contribute to cell communication in bone sarcomas [87].

Finally, the well-known immunosuppressive effects of MSCs can be mediated by their EVs, as suggested by recent results of a clinical trial involving patients with chronic kidney disease [88] and in preclinical model of osteoarthritis [89]. MSC-secreted EVs bearing TGF-β-induced gene product-h3 (TGFβI/BIGH3) could ameliorate osteoarthritis, however they can worsen tumor development by suppressing the activity of immune cells.

## 4. Vascular Microenvironment in OS Biology

### 4.1. Physiologic Angiogenesis during Bone Development

During both normal development and regenerative processes, blood vessels are major actors in bone biology, providing all necessary nutrients and oxygen to the highly dynamic bone tissue [90]. Beside their role as an efficient transport network for molecules and hematopoietic cells, blood vessels also support bone formation and homeostasis [91]. In long bones, the vasculature has a unique complex architecture, composed of a set of arteries penetrating the cortical zone and forming a heterogeneous network of capillaries, drained by a central vein [92]. New insights into the anatomical architecture and physiology of the vascular system in long bones have emerged with the description of trans-cortical microvessels originating in the bone marrow and forming connections between the endosteal and periosteal circulation [93]. Recently, a new bone-specific capillary subtype was identified in mouse juvenile tibia [94]. It is characterized by type-H endothelial cells that are rich in CD31 ^high^ Endomucin^high^ and are specifically localized close to the growth plate in the metaphysis and in the endosteal regions. During bone growth and modeling, these type-H cells have high proliferative capacities and are responsible for promoting the bone angiogenesis required for long bone growth. Furthermore, these type-H capillaries provide a favorable niche for Osterix^+^ osteoprogenitors, as they couple angiogenesis to osteogenesis through hypoxia-induced secreted Notch-ligand Noggin [94,95]. These populations of endothelial cells, the amount of which decreased with age in mice, have also been described in humans and have been proposed as an indicator of bone loss [96]. The roles of these bone-specific endothelial populations still needs to be elucidated in the context of bone tumor angiogenesis.

### 4.2. Neo-Vascularization in OS

Vascularization is an essential factor for tumor growth and dissemination, providing oxygen and nutrients, and supporting intravasation and extravasation of cancer cells. Elaboration of a tumor-dedicated vascular environment is one of the main hallmarks of cancer [97], and the formation of a tumor vascular network relies mainly on tumor piracy of physiological sprouting neo-angiogenesis mechanisms from existing vessels. Nevertheless, additional processes like vasculogenesis and vascular mimicry may also contribute to the expansion of a tumor vascular network [98].

Tumor angiogenesis is initiated by environmental stresses (hypoxia, acidosis) leading to disequilibrium of the pro-/anti-angiogenic balance, and consequently to the elevated expression of pro-angiogenic factors such as hypoxia-induced factor (HIF) and VEGF. OSs are highly vascularized bone tumors, lying in a hypoxic and acidic bone microenvironment. How neovascularization occurs in OS remains poorly understood. Nevertheless, OSs appear preferentially in the region of bone growth close to the metaphysis, where type-H endothelial cells promoting angiogenesis are located [94,95], suggesting a possible implication of these cells in OS neo-angiogenesis. In tumors, it is commonly admitted that neo-angiogenesis sprouting from pre-existing vessels is the most relevant process of angiogenesis, but the implication of endothelial progenitors cells (EPCs), able to differentiate into mature endothelial cells, has to be mentioned in tumor vascular growth, even if there is less of a consensus on these cells’ involvement [99]. EPCs may be recruited from their initial location in the bone marrow to the tumor site, or EPCs may reside in the tumor microenvironment, where tumors and stromal cells can provide plasticity and differentiation paracrine signals to EPCs. In Ewing sarcoma bone tumor, bone marrow EPC-induced vasculogenesis has been identified as an essential step of neovascularization and is crucial for tumor growth, with 10% of neo-vessels containing bone-marrow-derived cells [100]. In OS, a role for EPCs in neovascularization was also recently highlighted in an in vitro rat model, where co-injection of encapsulated OS cells with EPCs enhanced tumor vascularization [101]. In that case, OS rat tumor cells promoted migration and angiogenic properties of EPCs, through their specific secretome of angiogenesis-related factors (VEGF, TGF-β1, MCP-1, Activin A, and OPN).

Initially described in uveal melanoma, vascular mimicry, an alternative process to angiogenesis and vasculogenesis, is characterized by the de novo formation of perfusable, matrix-rich, vasculogenic-like micro-channels generated by transformation of aggressive tumor cells [102,103]. OS tumors appear to develop such an endothelial-free tumor-derived vasculogenic network, as vascular mimicry is found in 22.7% of osteoblastic-type OS samples and associated with unfavorable prognostis [104,105]. Human OS cell lines (U2OS [106], MG-63 [105]) and aggressive canine cell lines [107] are able to form vessel-like structures in three-dimensional cultures. Vascular mimicry mechanisms remain largely unknown, often being assessed via inappropriate and biased in vitro assays [108]. Nevertheless, a subpopulation of OS tumor cells co-expressing high levels of both VEGF and its receptor VEGFR-1 has been described as an important factor of aggressiveness [109]. This autocrine VEGF/VEGFR-1 signaling, associated with increased tumor growth and tumor vascularity, may possibly confer to OS cells the capacity to develop vasculogenic properties, leading to vascular mimicry. In fact, silencing *VEGF* suppresses vasculogenic mimicry in OS in vitro [110].

For many years, pro-angiogenic factors like VEGFs and angiopoietins have been considered paracrine soluble factors secreted by tumor cells and measurable in patient serum. However, EVs now appear to be essential players of intercellular communication, especially in tumors and in particular in the dialogue promoting angiogenesis. Indeed, stimulation of angiogenesis by tumor-derived EV cargo has been highlighted in numerous tumors [111]. In the context of OS, two recent studies established the pro-angiogenic role of OS-EVs through their cargo containing angiocrines and angiogenesis-related miRNAs [112,113].

### 4.3. Vascular and Angiogenic Factors in OS Patients

Several analyses of cohorts of OS patients have revealed the importance of neo-vascularization markers in patient samples. Amplification of genes in the VEGF pathway, in particular *VEGF-A*, has been described in OS patients, and was confirmed at the protein level [114]. Expression of high VEGF is positively associated with tumor stages and with metastasis [115,116]. Accordingly, a significant increase in vascularity density appears to be a hallmark of primary OS tumor in metastatic vs. non-metastatic patients [117]. Indeed, several clinical studies correlated high expression of VEGF in biopsies with worse disease-free survival and lower overall survival either in untreated [115] or in pre-operative treated patients [118]. Along these lines, a systematic review issued from a meta-analysis including 559 patients from 12 retrospective studies suggested that VEGF expression could be considered an effective biomarker of prognosis on OS patients [119]. On the other hand, conclusions drawn from another meta-analysis [120] underlined the importance of considering heterogeneity and geographic origin of patients. Beside VEGF, the expression of its receptor VEGFR-2 is increased in OS as compared to normal bone tissues, and high VEGFR-2 expression is associated with poor prognosis [121]. Investigation of angiogenic circulating factors also revealed that serum concentration of VEGF in bone sarcoma (Ewing sarcoma, OS, chondrosarcoma) was higher than in healthy samples or benign tumors [122]. The prognostic value of circulating VEGF was addressed in one study [123], as patients with metastasis at diagnosis or within a year of diagnosis had significantly higher serum levels of VEGF than non-metastatic patients without early metastatic disease. Level of circulating VEGF was independent of the tumor size and serum concentrations of two angiogenic factors, FGF2 and placental growth factor (PGF) were not different in metastatic vs. non-metastatic stage patients. Therefore, considering tissue or circulating angiogenic factors as objective prognostic factors still needs to be evaluated in larger prospective studies.

### 4.4. Targeting the Vascular Microenvironment with Anti-Angiogenic Agents

Targeting neo-vascularization has been considered in OS, with several Phase I/II clinical trials conducted during the past decade, mainly in advanced-stage OS [124]. The most promising clinical trials have been those including anti-angiogenic agents targeting VEGFRs, such as sorafenib alone [125] or associated with everolimus, an inhibitor of mTOR2 [126]. These trials published by the Italian Sarcoma Group in recurrent OS showed clinical benefits with an increase in 4–6 months progression-free survival for 43–46% of patients. On the other side, associating the well-described anti-VEGF antibody bevacizumab with pre- and post-operative chemotherapies in OS patients with resectable and localized OS did not improve the percentage of good responders to neo-adjuvant therapy, nor did it improve the outcomes of patients. Nonetheless, the treatment could induce post-surgical wound healing troubles [127]. In one case of spinal OS, association of sorafenib with the antibody denosumab, targeting RANKL present in the osteoid matrix, led to an efficient metabolic tumor regression [29]. A recent anti-angiogenic therapeutic strategy explored in preclinical studies is the targeting of VEGFR-2, a VEGF receptor expressed mainly on angiogenic vessels but also on OS tumor cells [121]. Results showed that the highly specific VEGFR-2 inhibitor, apatinib, was associated with direct anti-tumoral activity through VEGFR2/STAT3/BCL2 signaling [121,128]. Targeting VEGFR-2 with the monoclonal antibody ramucirumab was also shown to exert anti-angiogenic activity in vitro [129]. Surprisingly, injection of anti-mouse Vegfr-2 antibody in preclinical OS pediatric cancer models did not affect tumor growth, even when combined with cytotoxic chemotherapy using doxorubucin, suggesting that targeting both tumor and vascular microenvironment is mandatory in OS, even if it is difficult to achieve efficiently.

## 5. Cells of the Immune System in OS Microenvironment

The immune context of the OS microenvironment is mainly composed of tumor-associated macrophages (TAMs), with a significant number of dendritic, lymphoid, and myeloid cells [130]. Indeed, both myeloid and lymphoid cells have been detected in OS [130,131]. In contrast to the majority of epithelial tumors [132,133], studies have shown that TAMs were present in the immune infiltrate in a high proportion of biopsies and that an increased infiltration was associated with reduced metastasis and improved survival in high-grade OS [117,134,135]. The mechanism by which TAMs inhibit metastasis in OS remains unclear, but the observation by different teams that OSs were infiltrated with a heterogeneous population of pro-inflammatory M1 and anti-inflammatory M2 TAMs may suggest that in this precise case, the constitutive presence of M2 macrophages may have an anti-metastatic rather than a pro-metastatic effect [134]. Based on these data, OS patients were enrolled in a randomized clinical trial using the macrophage-activating agent mifamurtide in addition to a standard chemotherapy regimen, resulting in a significant improvement in 6 year overall survival [136,137]. Similar results were obtained in earlier studies in human and canine OS [138,139,140]. Mifamurtide has thus become the first new therapeutic drug used for the treatment of OS in the last 20 years [137]. More recently, the SARCOMA13 clinical trial (NCT03643133) proposed the use of the combination of mifamurtide with conventional chemotherapy in the treatment of French OS patients after surgery.

The observation that cytotoxic CD8 T lymphocytes are less abundant than myeloid cells in OS biopsies suggests that OSs are poorly immunogenic tumors, with a lack of tumor neo-antigens and scarce infiltrate of immune cytotoxic lymphocytes. This would define OSs as cold tumors, characteristic of pediatric tumors [141]. In recent studies, the presence of CD8 T lymphocytes was described in half of OS patient samples at diagnosis, and their presence was significantly associated with a lower rate of metastasis [135,142]. In addition, the ratio of intra-tumor CD8 T cells to regulatory immunosuppressive FOXP3 CD4 T cells in initial biopsies allowed discrimination of OS patients with prolonged survival from non-survivors [142]. As for most other tumors, OS infiltration by antigen presenting cells (APCs), including CD1a dendritic cells (DCs) and CD68 macrophages, has been correlated with poorer prognosis. Moreover, tumor programmed death-ligand 1 (PD-L1) expression has been associated with a poorer 5 year event-free survival [143]. Programmed death 1 (PD-1) is expressed at the surface of activated CD8 T lymphocytes, B lymphocytes, and natural killer (NK) cells. The involvement of the PD-1/PD-L1 checkpoint in OS varies from one study to another. In a recent systematic meta-analysis of 14 studies with a total of 868 patients, OS patients had 14–75% higher PD-L1 expression in tumor tissues, which was significantly correlated with metastasis, mortality risk, and poorer overall survival [144,145]. However, PD-L1 staining was detected in few OS specimens in two independent studies [135,146], which suggested that the role of the PD1/PD-L1 checkpoint is not predominant in the pathogenesis of OS. Nevertheless, several clinical trials using targeting PD-1/PD-L1 have been proposed. Among them, only one using anti-PD-L1 avelumab (NCT03006848) is recruiting, while three trials using anti-PD-1, penbrolizumab (NCT02301039 and NCT03013127), or nivolumab (NCT02304458) are either not recruiting or suspended, likely due to risk of immune-related side effects such as skin reactions, pneumonitis, colitis, and hepatitis [147,148].

With the extraordinary clinical success of adoptive therapies based on the use of chimeric antigen receptor (CAR) engineered T cells in B-cell malignancies [149], attempts to develop such therapeutic strategies are emerging in solid tumors. In the context of OS, CAR-T-cell therapy still remains largely unexplored. The main challenge of this immune approach is the identification of an optimal target expressed restrictively on tumor cells, in order to avoid toxicity and to selectively eradicate cancer cells. Up to now, few potential targets have been identified in OS [150]—the Human epidermal growth factor 2 (HER2), the Insulin-like Growth factor receptor-1 (IGFR1), and the tyrosine orphan-like receptor 1 (ROR1). HER-2 CAR-T cells have been shown to drive tumor regression in an animal model [151]. Tested in a phase I/II clinical trial in HER-2 positive sarcoma patients, infusion of HER-2 CAR-T was shown to induce no toxicity, and the 6 week stability of these cells [152] paved the way for further trials. Preclinical studies highlighted that IGFR1 and ROR1 that are overexpressed in OS cells lines are also relevant targets. An adoptive transfer of IGFR1-CAR and ROR1-CAR T cells derived from a sarcoma patient significantly suppressed tumor growth in both localized and disseminated sarcoma xenograft models [153]. In OS, the main issues with CAR-T cells would be accessing their target through a stiff osteoid bone tumor matrix, allowing their survival in a local immunosuppressive microenvironment. Despite promising preclinical results, launching the use of CAR-T cell therapies in OS is still a matter of debate.

## 6. The Multi-Kinase Inhibitors (MKIs) as Promising Therapies in OS Treatment

Targeted therapies using MKIs proved their efficacy initially in renal cell cancer, hepatocellular carcinoma, and thyroid cancer [154]. These molecules exert an anti-cancer activity by simultaneously targeting several kinases. Initially developed as potent VEGFR signaling inhibitors, it is now admitted that their anti-tumor action depends on both the inhibition of angiogenic but also non-angiogenic pathways.

In the context of OS, several in vitro and preclinical results have highlighted the relevance of MKIs as potential therapeutic tools. Anlotinib, a VEGFR2 and MET kinase inhibitor, suppressed tumor growth and metastasis and increased chemosensitivity of OS cells [155]. Moreover, cabozantinib, an inhibitor of c-MET (also known as HGFR), VEGFR-2, c-KIT, and TIE-2, appears to block proliferation and migration of OS cells via autophagy. Additionally, it acts on the bone microenvironment by decreasing RANKL and increasing OPG production by osteoblasts [156], which results in a strong inhibition of RANK-positive OS cell proliferation [157]. Exploration of MKIs in clinical trials (Table 1) started initially with sorafenib [126], as described above. Subsequently, a therapeutic strategy using regorafenib, an MKI that targets angiogenic (VEGFR1-3, TIE-2), stromal (PDGFR-β, FGFR), and oncogenic kinases (KIT, RET, and RAF) has been tested. Notably, two clinical trials [158,159] were conducted in patients with recurrent, progressive, metastatic OS after failure of conventional chemotherapy and demonstrated a significant extension of progression-free survival. These studies highlighted the potential therapeutic value of MKIs in very aggressive and resistant OS. These very promising results paved the way for further exploration of this type of targeted therapy. In this context, pazopanib, a MKI inhibiting VEGR1-3, PDGFRα/β, and c-KIT, showed objective responses in metastatic or relapsed patients [160,161]. Preclinical and in vitro data reinforced the relevance of MKI in OS. One phase II multi-centric clinical trial has just been published, exploring cabozantinib in advanced relapsed OS and Ewing sarcoma [162]. Encouraging responses were reported for both pathologies. In OS, 12% patients presented a 6 months objective response and 33% patients had no progression in 6 months. Additionally, a phase II clinical study in relapsed refractory OS (NCT04154189) starting in February 2020 involves lenvatinib, a MKI inhibiting VEGFR1-3, FGFR1-4, PDGFRα, KIT, and RET and used in thyroid cancer [163].

## 7. Discussion

In the past decade, the supportive role of the bone microenvironment in the tumor progression and metastatic dissemination of OS has been intensively documented. This culminates in the characterization of a vicious cycle established between OS cells and osteoclasts, where tumors, by promoting osteoclastic activity, indirectly lead to the degradation of bone matrix and to the release of pro-tumor factors initially trapped in the bone matrix.

Intuitively, targeting the bone resorption in order to limit tumor growth thus appeared to be a promising therapeutic option. Therefore, the bisphosphonate ZOL was the first anti-resorptive agent to be explored as a combined treatment for OS. Unfortunately, its translation into the clinical setting was abandoned due to disappointing results obtained in the clinical trial OS2006, since it showed no benefit for OS patients [25]. Along the same line, blocking osteolysis by using denosumab, a humanized antibody directed against RANKL, is no longer considered a potential combined treatment for OS patients. Indeed, if RANK signaling transduces signal in different cell types, including osteoclasts, stromal cells, endothelial cells, and dendritic cells, it is not directly implicated in OS cell division and survival. Importantly, the description of the reverse RANK-RANKL signaling, involving membrane RANKL activation by RANK-bearing EVs, is a new and unexpected regulatory pathway of bone remodeling that has to be considered. Such bidirectional regulation occurring simultaneously in osteoblasts and osteoclasts within a bone multicellular unit has been previously described for Ephrin membrane ligands and their receptors [164]. Therefore, the reverse RANK-RANKL signaling should be explored in other cell types and pathological conditions including OS. Moreover, new anti-RANKL antibodies should be produced to inhibit osteoclasts without effect on osteoblasts.

EVs have now to be considered as key actors of cellular communication in the OS tumor microenvironment ecosystem. EVs originate from the tumor itself, but also from MSCs and osteoclasts. EVs appear to be essential for mediating signals for bone remodeling, angiogenesis, and also for immune responses. EVs modulate gene expression by transferring their cargo to cells and drive communication between cells through many combinations. As reported in this review, EVs are able to transfer growth factors, cytokines, and nutrients. In addition, they can also transfer nucleic acids. Notably, DNA can be expelled from cells by EV release during drug-induced senescence in order to maintain cellular homeostasis [86], whereas microRNAs contained in EVs can contribute to modulating cell communication in bone sarcomas [87]. Blocking EV-mediated signaling would be an appealing strategy to counteract deleterious and pro-tumor communications in the OS environment. Additionally, EVs are currently being explored as potential vectors of therapies but not yet as targetable entities [165].

Targeting the immune system by the macrophage-activating agent mifamurtide resulted in the most recent improvement of OS therapy since polychemotherapy. However, stimulating the immune system by using antibodies directed against PD-1/PD-L1 in OS is still debated. This debate includes toxic effects of antibodies, poor CD8 T lymphocytes infiltrating OS, no histological evidence of the PD-1/PD-L1 in most OS samples, and no correlation with tumor outcome to date [135].

With the same logic of hitting the microenvironment in order to block tumor progression, targeting the vascular environment, which is particularly developed and supportive in OS, has also been evaluated. If the first clinical trial including the anti-VEGF antibody bevacizumab was disappointing [127], encouraging results emerged from initial clinical trials using the MKI sorafenib, which targets intracellular kinase activity of VEGFRs [125,126]. Since then, several MKIs like regorafenib [158,159], pazopanib [160,161], and more recently cabozantinib, have been reported to have beneficial effects in advanced OS and Ewing sarcoma bone tumors [162]. Accordingly, these agents had promising therapeutic results in selected patients, probably through the combined action on both angiogenic vascular compartment and OS cells in which they inhibit multiple growth factor pathways with potential oncogenic activity (c-MET, c-KIT).

In conclusion, the MKI class of small molecules represents a significant major advance in OS therapy in recent decades, and reinforces the notion that considering OS in its whole ecosystem and thereby targeting several of its components, including EVs, is the way to successful therapies.

## Figures and Tables

**Table 1:** Multi-kinases inhibitors (MKI) in Osteosarcoma (OS): Molecular and Cellular Targets and Clinical Trials.

MKI	Targets		Clinical Trials
	Molecular	Cellular	ClinicalTrials.gov
Sorafenib	RAF, KIT, FLT3, RET VEGFR1-3 PDGFRβ	Tumor Endothelial Stromal	NCT 00889057 [125] NCT 01804374 [126]
Regorafenib	KIT, RET, RAF VEGFR1-3, Tie-2 PDGFRβ FGFR	Tumor Endothelial Stromal	NCT 0238244 [159] NCT 02048371 [158]
Pazopanib	KIT, FMS VEGFR1-3 PDGFRαβ FGFR	Tumor Endothelial Stromal	[160]
Cabozantinib	MET, KIT, RET VEGFR-2, Tie-2	Tumor Osteoblasts Endothelial	NCT02243605 [162]
Lenvatinib	KIT, RET VEGFR-1, 2, 3 PDGFR-α FGFR1-4	Tumor Endothelial Stromal	NCT04154189
